# Prevalence of *E. coli* ST131 among Uropathogenic *E. coli* Isolates from Iraqi Patients in Wasit Province, Iraq

**DOI:** 10.1155/2020/8840561

**Published:** 2020-10-16

**Authors:** Dhifar Raa'd Al-Guranie, Sareaa Maseer Al-Mayahie

**Affiliations:** Microbiology, Department of Biology, College of Science, Wasit University, Kut, Iraq

## Abstract

The emergence of *Escherichia coli* sequence type 131 (*E. coli* ST131) clone represents a major challenge to public health globally, since this clone is reported as highly virulent and multidrug-resistant, thus making it successfully disseminated worldwide. In Iraq, there is no previous study dealing with this important clone, so this project was suggested to investigate its presence within uropathogenic *E. coli* (UPEC) from Iraqi patients in Wasit Province. A total of 112 UPEC isolates from cases of acute urinary tract infection (UTI) were analysed for phylogenetic groups by quadruplex PCR; then, these isolates were investigated for *E. coli* ST131 clone by both conventional and real-time PCR procedures. The antibiotic susceptibility test was performed by the disk diffusion method. The results revealed that, out of 112 UPEC isolates, 38 (33.9%) belonged to phylogroup B2. For conventional PCR, 92.1% (35/38) of B2 *E. coli* isolates were positive for *E. coli* ST131, of which 34 were O25b-ST131 strain and 1 was O16-ST131 strain. However, serogroups O25b and O16 represented 17.1% and 2.8%, respectively. By RT-PCR assay, 15.1% (17/112) and 44.7% (17/38) of total and B2 *E. coli* isolates were confirmed as being *E. coli* ST131, respectively. The highest resistance rates of *E. coli* ST131 isolates were against the *β*-lactams, while low resistance rates were against amikacin, nitrofurantoin, and gentamicin. Fortunately, all isolates were susceptible to carbapenems. Moreover, 52.9% (9 out of 17) of *E. coli* ST131 isolates were MDR. In conclusion, the presence of *E. coli* ST131 among UPEC isolates from Iraqi patients is confirmed with high resistance to most antimicrobials included in this study.

## 1. Introduction

Urinary tract infection (UTI) is a common health problem in both community and nosocomial settings [1]. *Escherichia coli* (*E. coli*) is one of the most important causes of UTI, whether it is hospital-acquired or community-acquired [2]. The emergence of *E. coli* sequence type 131 (ST131) as a multidrug-resistant (MDR) and virulent pathogen represents a major challenge to public health globally [3].

*Escherichia coli* ST131 is the predominant extraintestinal pathogenic *E. coli* (ExPEC) that belongs to the highly virulent phylogenetic group B2, and its strains are mostly of serotype O25:H4, with a specific O25 type, O25b [3]. The production of extended-spectrum *β*-lactamases (ESBLs) in the Enterobacteriaceae family has increased significantly, especially in *E. coli* ST131 clone, which is strongly associated with ESBLs [3, 4]. A single ExPEC clone, ST131, is predominantly responsible for global fluoroquinolone resistance (FQ-R) and cephalosporin resistance (ceph-R) pandemic, causing millions of antimicrobial-resistant infections annually (e.g., up to 30% of all ExPEC, 60–90% of FQ-R ExPEC, and 40–80% of ESBL ExPEC belongs to ST131) [5]. Thus, *E. coli* O25b-ST131 clone shows a high resistance profile to many drugs and this leaves a few effective antibiotic options that can be used to treat patients [2]. Plasmids represent a major vehicle for the carriage of antibiotic resistance genes [6], and their association with widespread successful *E. coli* clones, such as the pandemic O25b-ST131 clone, further facilitates their dissemination by clonal expansion [7]. The increasing prevalence of MDR Enterobacteriaceae limits available treatment options for infections caused by these organisms [8]. In Iraq, to our knowledge, a survey of the literature showed no information on *E. coli* ST131 distribution. Hence, this project was presented to detect this clone's prevalence among UPEC isolates from outpatients with acute UTI in Al-Kut City, Wasit Province, Iraq, by conventional and real-time PCR procedures.

## 2. Materials and Methods

### 2.1. Bacterial Isolates

In the present study, of 112 UPEC isolates, 32 UPEC isolates were collected from outpatients with acute UTI and 80 UPEC isolates were obtained from Microbiology Laboratory at the Department of Biology, College of Science, Wasit University.

### 2.2. Survey of UPEC for *E. coli* ST131 Clone

At first and according to methods provided by Clermont et al. [9], *E. coli* isolates were screened for their reference to one of the eight *E. coli* phylogenetic groups (A, B1, B2, C, D, E, F, and clade I) based on the presence of *chuA*, yjaA, and *arpA* genes and *TspE4.C2* DNA fragment (Table 1). Then, all B2 *E. coli* isolates were screened for *E. coli* ST131 by conventional multiplex PCR assay (Table 2), which combines the identification of O25b and O16 ST131 clades using the primer sets and PCR conditions described by Johnson et al. [10]. Thereafter, these B2 isolates were surveyed by RT-PCR for *E. coli* O25b-ST131 clone according to Dhanji et al. [11] (Table 3).

### 2.3. Antimicrobial Resistance of *E. coli* ST131 Isolates

Antimicrobial resistance of *E. coli* ST131 isolates was performed by the disk diffusion method according to the instructions of CLSI [12]. The used antimicrobials were ampicillin (AMP: 10 *μ*g); amoxicillin-clavulanic acid (AMC: 20/10 *μ*g); cefoxitin (FOX: 30 *μ*g); cefotaxime (CTX: 30 *μ*g); ceftazidime (CAZ: 30 *μ*g); ceftriaxone (CRO: 30 *μ*g); cefepime (FEP: 30 *μ*g); aztreonam (ATM: 30 *μ*g); imipenem (IPM: 10 *μ*g); meropenem (MEM: 10 *μ*g); gentamicin (CN: 10 *μ*g); amikacin (AK: 30 *μ*g); tetracycline (TE: 30 *μ*g); nalidixic acid (NA: 30 *μ*g); ciprofloxacin (CIP: 5 *μ*g); trimethoprim-sulfamethoxazole (SXT: 1.25/23.75 *μ*g); and nitrofurantoin (F: 300 *μ*g).

## 3. Results

### 3.1. Phylogenetic Groups of UPEC

**Figure 1:**
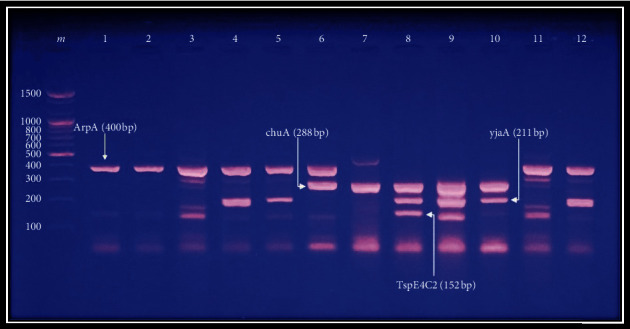
Agarose gel (2%) electrophoresis (1 volt/5 cm) of ethidium bromide-stained products (genes: *Arp*, *chuA*, *YiaA*, and *TspE4C2*, respectively) of quadruplex PCR profiles of representative *E. coli* isolates. Lane m: DNA ladder (100 bp); lanes 1 and 2: group A (+ − − −); lanes 3 and 11: group B1 (+ − − +); lane 4: group A (+ − + −); lane 5: group C (+ − + −); lane 6: group D (+ + − −); lane 7: group F (− + − −); lanes 8 and 9: group B2 (− + + +); lane 10: group B2 (− + + −); and lane 12: group C (+ − + −).

Phylogenetic analysis revealed that the highest frequency of this study's isolates was in group B2 (33.9%), followed by group A (24.1%), group D (17.85%), group B1 (8.03%), group C (4.4%), and group F (3.5%). Group E was not detected in any isolate, and 9 isolates (8.03%) were nontypeable (Figure 1).

### 3.2. Molecular Detection of *E. coli* ST131 Clone

**Figure 2:**
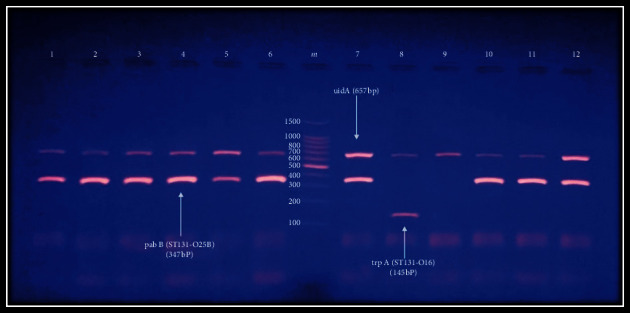
Electrophoresis (1 volt/5 cm) of ethidium bromide-stained agarose gel (2%) of PCR-amplified products for detection of representative *E. coli* ST131 clone. Lane m: DNA ladder (100 bp), upper band corresponds to the amplification of the internal control *uidA* gene (657 bp); lanes 1–6, 7, and 10–12: positive results for allele-specific amplification of the ST131-O25b clade *pabB* (347 bp); lane 8: positive result for ST131-O16 clade *trpA* variants (145 bp); lane 9: represents non-ST131 *E. coli*.

**Figure 3:**
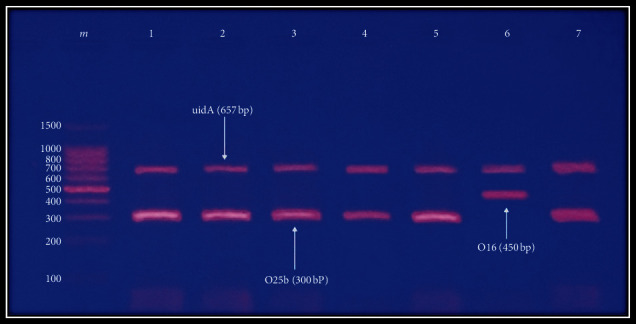
Electrophoresis (1 volt/5 cm) of ethidium bromide-stained agarose gel (2%) of PCR amplified products for detection of representative *E. coli* ST131-O types. Lane m: DNA ladder (100 bp), upper band corresponds to the amplification of the internal control *uidA* gene (657 bp); lanes 1, 2, 3, 4, 5, and 7: positive results for O25b rfb (300 bp); lane 6: positive results for O16 region (450 bp).

The existence of *E. coli* ST131 clone is restricted to phylogroup B2 [2,13,14]. Therefore, in this research, only isolates in phylogroup B2 (*n* = 38) were investigated for this clone by both conventional and RT-PCR procedures. For conventional PCR, 92.1% (35/38) of B2 *E. coli* isolates were positive for *E. coli* ST131 clone, of which 34 were O25b-ST131 strain and 1 was O16-ST131 strain (Figure 2). However, serogroups O25b and O16 represented 17.1% and 2.8%, respectively (Figure 3). To be sure of the procedures' specificity, it was applied to representative *E. coli* isolates belonging to other phylogroups. The results revealed that 100% of isolates in groups B1, D, C, and E and 50% and 33.3% of isolates in groups A and NT, respectively, were positive for *E. coli* O25b-ST131. Of these isolates, only one isolate (14.8%) belonged to group B1 was positive for serotype O25b (Table 4).

**Figure 4:**
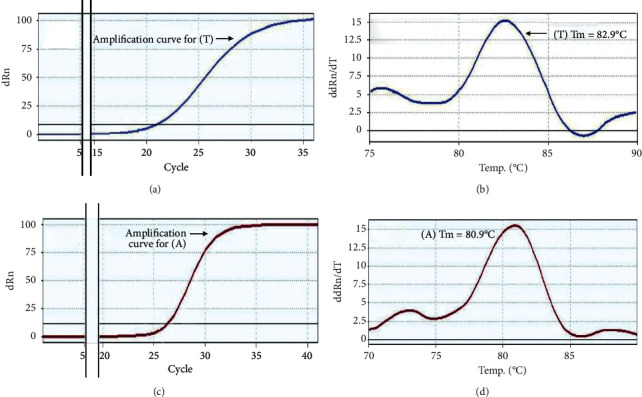
RT-PCR detection of representative *E. coli* clone ST131 allele by analysis of amplicon melt curves. (a, c) Amplification curves for T-SNP and A-SNP expressions depending on SYBR Green I RT-PCR, respectively. (b) Thymine “T” assay for *pabB* with primers ST131 (TF/TR) to identify T144 single-nucleotide polymorphism (SNP); melting temperature (Tm: 82.9°C) = ST131 *E. coli.* (d) Adenine “A” assay for *pabB* with primers ST131 (AF/AR) to identify A450 single nucleotide polymorphism (SNP); melting temperature (Tm: 80.9°C) = ST131 *E. coli*.

By RT-PCR assays, 44.7% (17/38) of B2 *E. coli* isolates were confirmed as being *E. coli* ST131 clone, where they showed positive results for both “A” and “T” SNP specific for ST131 clone with a mean ± standard deviation (SD) Tm value of 82.82 ± 0.08°C and 80.69 ± 0.09°C in the Thymine “T” i and Adenine “A” SNP real-time PCR assays, respectively (Table 5 and Figure 4). In addition, 3 (42.8%) isolates of phylogroup B1 gave positive results with a Tm of 82.82 ± 0.08°C in the thymine “T” SNP assay, but negative results with a Tm of 80.69 ± 0.09°C in the adenine “A” SNP assay.

All *E. coli* ST131 isolates (*n* = 17) were tested for their resistance to 17 antimicrobials belonging to different classes. The highest rates of resistance were against the *β*-lactams except for carbapenems to which all of the isolates were susceptible. In addition, there were high resistance rates against tetracycline, trimethoprim-sulfamethoxazole, nalidixic acid, and ciprofloxacin, while low resistance rates were observed against amikacin, nitrofurantoin, and gentamicin. Moreover, 9 (52.9) of *E. coli* ST131 isolates were MDR (Table 6).

## 4. Discussion

Of 112 UPEC isolates, 38 (33.9%) belonged to phylogroup B2. This high prevalence of group B2 among UPEC isolates obtained in this study is in agreement with what it is well known worldwide, such as studies conducted by Iraqi researchers [1, 15, 16]. In addition, studies from different countries [14,17–19] indicated that this group was the most prevalent among UPEC isolates. This predominance of group B2 among UPEC isolates may be attributed to the fact that most virulence factors and antibiotic resistance genes existed jointly within this group and this could increase survival fitness in the urinary tract as recognized by many researchers [17, 20, 21].

Group B2 isolates were screened for *E. coli* ST131 as the presence of this clone is restricted to this group [2, 13, 14]. By conventional PCR, 92.1% of B2 isolates were *E. coli* ST131, whereas only 44.7% were found to belong to this clone by RT-PCR. Furthermore, members of other *E. coli* groups showed positive results for *E. coli* ST131 by conventional PCR, while none of them were positive for this clone by RT-PCR. These results indicated the occurrence of false-positive results with conventional PCR, which means that this procedure is useless for the detection of this clone, especially if we deal with large numbers of isolates for which long periods of time are required (i.e., storage of primers in the laboratory for long periods). The occurrence of false-positive results with conventional PCR was admitted by the developers of these procedures themselves who emphasized the possibility of false-positive results with allele-specific PCR, which depends on detecting the same SNPs, 3′-end base degradation of the primers, or horizontal gene transfer can give false-positive results [10, 22]. In addition, a significant challenge is that the sensitivity of PCR can easily result in contamination and consequently false-positive PCR results by cross-contamination between samples, cross-contamination between nucleic acids, and PCR product carryover contamination [23]. Therefore, RT-PCR was carried on as it is the most accurate in determining *E. coli* ST131 according to Dhanji et al. [11], who used together the thymine “T” and adenine “A” ST131-specific *pabB* SNP assays, and does not require post-PCR product handling, preventing potential PCR product carryover contamination [23].

The only study in which *E. coli* ST131 clone was detected in Iraq was that achieved by Al-Hilali [24] in Najaf Province, where he did not find any isolate belonging to the O25b-ST131 clone among MDR UPEC isolates from patients with significant UTI in Najaf hospitals. This may be due to the low percent (1.1%) of B2 isolation in his study. This prevalence of *E. coli* ST131 (15.1% and 44.7% of total and B2 *E. coli* isolates, respectively) among UPEC isolates from Iraqi patients included in this study was in accordance with what is globally known by other researchers [ 2, 3, 17, 19, 25], where this clone is successfully disseminated worldwide, and it has become the most predominant lineage associated with a variety of infections around the globe [2, 17]. This high dissemination may be due to several reasons, the most important one is the epidemic potential, and virulence and multidrug resistance ability, as well as its predominance in the human gut, make them rapidly disseminated worldwide, with a high capability of causing extraintestinal infections, particularly UTIs. So that, the dominance of this clone was higher among EXPEC [2, 25– 27]. Furthermore, diverse modes of transmission of resistance, probably with a major role of plasmids, encourages the worldwide spread of this clone [28]. Several studies have investigated the important role of these plasmids, in addition to bacteriophages, in the widespread of this clone around the globe [29, 30] who suggested that this species is a generalist able to colonize and infect humans. Also, it was suggested that bacteriophages and plasmids make an important contribution to specialization by accessorizing the genome with new adaptive traits and tools that modify genome structure and, eventually, by modifying transcriptional regulation. Therefore, they have a tendency to acquire multidrug-resistant phenotypes and are difficult to treat [25].

Antimicrobial resistance in UPEC is a major concern worldwide due to its increased resistance to several antibiotics [31], particularly to fluoroquinolones, trimethoprim-sulfamethoxazole, and cephalosporins, which coincided with the emergence of ST131 [32]. Indiscriminate and widespread use of antibiotics in addition to the practice of prescribing antibiotics to treat UTI without bacterial characterization led to increased resistance among uropathogens and to decreased effectiveness of oral therapies [8,33], which gave an alarming level of antimicrobial resistance developing in UTI pathogens. Thus, rapid initiation of appropriate empirical treatment requires a good knowledge of epidemiological data concerning the sensitivity of uropathogens to antibacterial agents [34]. In this investigation, the highest rate of resistance in *E. coli* ST131 isolates was against *β*-lactams, tetracycline, trimethoprim-sulfamethoxazole, nalidixic acid, and ciprofloxacin which was comparable with the results of several studies around the globe [3,17,19,27,32,35,36], and this may be due to several reasons including inexpensive antimicrobials, indiscriminate antibiotic usage without medical prescription, and use of antibiotics for a nonoptimal duration. In addition, plasmids harboring resistance determinants can be transferred between bacteria, even between species, leading to the acquisition of resistance to new antibiotics via the emergence of mutant strains [4]. In addition, some bacteria produce multiple *β*-lactamases, which may reduce the efficiency of *β*-lactam/*β*-lactamases inhibitor combinations [33]. The rapid development of resistance to *β*-lactam antibiotics attributed to the emergence of extended-spectrum *β*-lactamases (ESBL) in the enteric bacteria [24]. This may be due to the excessive use of expanded spectrum cephalosporins (ESC) during clinical practice, where several studies have found a relationship between third-generation cephalosporins use and acquisition of ESBL-producing strains [37]. Therefore, the limited use of these antibiotics might be helpful to inhibit/avoid the emerging or spreading of multidrug-resistant Gram-negative bacteria [38].

Fortunately, all *E. coli* ST131 isolates in this study were sensitive to imipenem (IPM) and meropenem (MEM), which present a broad spectrum of antibacterial activity and fewer adverse effects [39]. Similar results have been reported in other studies [3, 19]. These antibiotics are considered as the most reliable last-resort treatment for bacterial infections [39]. Thus, we noticed the high effectivity of these antibiotics against *E. coli* ST131 clinical isolates.

In addition, the low resistance rates against aminoglycosides (gentamicin: 17.6% and amikacin: 5.8%) reported in the present study may be attributed to the rare use of these antibiotics in AL-Kut hospitals which may be due to their high costs in comparison with *β*-lactams [37]. Similar resistance results of *E. coli* ST131 clone against gentamicin and amikacin (33.3%, 2.8%; 30%, 5%; and 44.2%, 10.4%, respectively) were revealed by Namaei et al. [3], Ali et al. [17], and Hojabri et al. [26], respectively. Nitrofurantoin resistance was also noted in 5.8% of *E. coli* ST131 clinical isolates, and comparable frequencies (10%, 11.4%, and 6%, respectively) were reported by many researchers [17, 32, 38]. In addition, Nuesch-Inderbinen et al. [35] and Zhong et al. [19] found that all isolates (100%) were sensitive to nitrofurantoin. This high susceptibility of *E. coli* ST131 isolates to nitrofurantoin may be due to the lower frequency use of this drug as explained by Munkhdelger et al. [40]. These results suggest that nitrofurantoin may be effective in treating UTIs caused by *E. coli* ST131 [19].

Moreover, multidrug resistance (MDR) was demonstrated in 9 out of 17 *E. coli* ST131 isolates (52.9%). Several studies were in matching line with the present study [13, 19, 27, 28, 37]. This may be due to the fact that *E. coli* pathogens, particularly, *E. coli* ST131 clone, have developed resistance to every class of antibiotics introduced to treat human and animal infections [41], and these infections are particularly challenging to treat [17]. This study showed that multidrug resistance to three, four, five, and six antimicrobial classes were observed in 23.5%, 17.6%, 5.8%, and 5.8% of *E. coli* ST131 isolates, respectively (Table 7). However, resistance to one and two classes was noted in 5.8% and 41.1%, respectively. High resistance rate among MDR classes was against 3 classes of antimicrobials, while resistance to 5 and 6 classes was observed in only 1 isolate. Regarding resistance patterns of MDR ST131 clone, the present study revealed that 7 resistance patterns (Table 8) were observed among *E. coli* ST131 clinical isolates. Patterns 3 (resistance to 4 classes) and 6 (resistance to 3 classes) were the most common and were seen in two isolates (11.7%). Other patterns, ranging from resistance against 6 classes in pattern 1, 5 classes in pattern 2, 4 classes in pattern 4, to 3 classes in patterns 5, 6 and 7, were observed in only one ST131 isolate.

The increase of MDR strains in this study may be the result of several reasons including overuse of antibiotics through their prescription or self-medication without antibiotic sensitivity testing, as well as sharing an antibiotic between family members or friends. Careless usage of antibiotics is the most important factor that facilitates the development of MDR, which triggers the selection and distribution of antibiotic-resistant pathogens in clinical practice [24, 42]. To end this problem, Al-Hilali [24] and Lee et al. [4] suggested that the antimicrobial therapy should be tailored to each patient, taking into consideration the severity of disease, individual and local patterns of antimicrobial resistance, and the potential for collateral damage associated with antimicrobial use. Thus, appropriate antibiotic use is an essential component of any program to slow the emergence and spread of drug-resistant microorganisms in the health-care setting.

## 5. Conclusion

The relatively high prevalence of *E. coli* ST131 clone among UPEC isolates from Iraqi patients highlights the necessity to work seriously to control the spread of this clone in our community by following reasonable strategies all over the country. According to the results of this investigation, it is recommended to use aminoglycosides (amikacin and gentamicin) and nitrofurantoin for the treatment of UPEC including *E. coli* ST131, especially in the study area.

## Figures and Tables

**Table 1:** Components of 50 *μ*l PCR master mix and amplification conditions for detection of UPEC phylogroups.

PCR reaction	Sterile DW (*μ*l)	Primers	DNA (*μ*l)	Amplification conditions
Quadruplex	37	8 *μ*l: 1 *μ*l each of (i) chuA-1b and chuA-2 (ii) yiaA.1b and yiaA.2b (iii) TspE4C2.1b and TspE4C2.2b (iv) Acek.f and ArpA.r	5	(1) Initial denaturation at 94°C for 4 min (2) 30 cycles of (i) Denaturation at 94°C for 5 s (ii) Annealing at 57°C (group E) or 59°C (quadruplex and group C) for 20 s (iii) Extension at 72°C for 1 min (3) Final extension at 72°C for 5 min
Group E	41	4 *μ*l: 1 *μ*l each of (i) ArpAgpE.f and ArpAgpE.r (ii) trpBA.f and trpBA.r	5	
Group C	41	4 *μ*l: 1 *μ*l each of (i) trpAgpC.1 and trpAgpC.2 (ii) trpBA.f and trpBA.r	5	

**Table 2:** Components of 50 *μ*l PCR master mix (2 pools) and amplification conditions for the surveillance of *E. coli* ST131 clone by conventional PCR assay.

Pool no.	Sterile DW (*μ*l)	Primers	DNA (*μ*l)	Amplification conditions
1 (three genes)	39	*6* *μ*l: 1 *μ*l each of (i) trpA_ST131-O16_. f (ii) trpA_ST131-O16_. r (iii) pabB_ST131-O25_. f (iv) pabB_ST131-O25_. r (v) uidA.f (vi) uidA.r	5	(1) Initial denaturation for 4 min at 94°C (2) 30 cycles of (i) Denaturation for 5 s at 94°C (ii) Annealing for 20 s at 63°C (iii) Extension for 30 s at 72°C (3) Final extension at 72°C for 5 min

2 (three genes)	39	*6* *μ*l: (i) 2 *μ*1 gndbis.f (ii) 1 *μ*l rfbO16.r (iii) 1 *μ*l rfbO25b.r (iv) 1 *μ*l uidA.f (v) 1 *μ*l uidA.r	5	(1) Initial denaturation for 4 min at 94°C (2) 30 cycles of (i) Denaturation for 5 s at 94°C (ii) Annealing for 20 s at 59°C (iii) Extension for 30 s at 72°C (3) Final extension at 72°C for 5 min

**Table 3:** Components of 20 *μ*l PCR master mix (2 pools) and amplification condition for detection of *E. coli* ST131 clone by RT-PCR assay.

Pool	Sterile double DW (*μ*l)	Primers	DNA (*μ*l)	Amplification conditions
ST131T	13	1 *μ*l each of (i) ST131T.F (ii) ST131T.R	5	(1) Initial denaturation at 95°C for 5 min (2) 40 cycles of (i) Denaturation at 95°C for 5 s (ii) And annealing at 58°C for 10 s (iii) The fluorescence signal was measured at the extension step (iv) Following amplification, a melting curve was generated by heating the PCR product to 95°C with a ramp rate of 0.05°C/s
ST131A	13	1 *μ*l each of (i) ST131 A.F (ii) ST131 A. R	5	

**Table 4:** Distribution of *E. coli* ST131 clone among phylogroups of UPEC isolates from patients with acute UTI by conventional PCR assay.

Phylogroups	No. of *E. coli* isolates	No. (%) of *E. coli* ST131-positive isolates	No. (%) of *E. coli* O25 and O16-positive isolates	
			rfb 25	rfb 16
B2	38	35 (92.1)	6 (17.1)	1 (2.85)
B1	7	7 (100)	1 (14.8)	0
A	6	3 (50)	0	0
D	3	3 (100)	0	0
C	1	1 (100)	0	0
F	2	2 (100)	0	0
NT	3	1 (33.3)	0	0

**Table 5:** Distribution of *E. coli* ST131 clone among phylogenetic groups according to RT-PCR results. Antimicrobial susceptibility of *E. coli* ST131 clone.

Phylogroups	No. of isolates	No. (%) of isolates positive with ST131 “T” SNP assay (Tm = 82.82°C)	No. (%) of isolates positive with ST131 “A” SNP assay (Tm = 80.69°C)	^*∗*^No. (%) of isolates assigned ST131 based on both “A” and “T” SNP assays
B2	38	19 (50.0)	27 (71.01)	17 (44.7)
B1	7	3 (42.8)	0	0
A	6	0	0	0
D	3	0	0	0
C	1	0	0	0
F	2	0	0	0
NT	3	0	0	0

**Table 6:** Antimicrobial resistance of 17 *E. coli* ST131 isolates.

Class of antimicrobial agent	Antimicrobial agent	No. (%) of resistant *E. coli* ST131 isolates
*β*-Lactams	AMP	17 (100): *R* = 17, *I* = 0
	AMC	15 (88.2): *R* = 12, *I* = 3
	FOX	13 (76.4): *R* = 7, *I* = 6
	CTX	12 (70.5): *R* = 10, *I* = 2
	CAZ	13 (76.4): *R* = 10, *I* = 3
	CRO	9 (52.9): *R* = 9, *I* = 0
	FEP	14 (82.3): *R* = 9, *I* = 5
	ATM	10 (58.8): *R* = 7, *I* = 3
	IPM	0
	MEM	0
Aminoglycosides	CN	3 (17.6): *R* = 2, *I* = 1
	AK	1 (5.8): *R* = 0, *I* = 1
Tetracyclines	TE	13 (76.4): *R* = 10, *I* = 3
Fluoroquinolone	NA	7 (41.1): *R* = 7, *I* = 0
	CIP	6 (35.2): *R* = 5, *I* = 1
Trimethoprim	SXT	8 (47.0): *R* = 8, *I* = 0
Nitrofurans	F	1 (5.8): *R* = 1, *I* = 0
Multidrug resistance (MDR)		9 (52.9)

R: resistant; I: intermediate.

**Table 7:** Number of classes to which *E. coli* ST131 isolates are resistant.

No. of classes	Number (%) of *E. coli* ST131 isolates
1 class	1 (5.8)
2 classes	7 (41.1)
^*∗*^3 classes	4 (23.5)
^*∗*^4 classes	3 (17.6)
^*∗*^5 classes	1 (5.8)
^*∗*^6 classes	1 (5.8)

*∗*Multidrug resistance.

**Table 8:** Resistance patterns of MDR *E. coli* ST131 clone from patients with acute UTI.

Pattern no.	Resistance pattern	No. of classes	No. (%) of *E. coli* ST131 isolates
1	AMP, AMC, CTX, CAZ, CRO, FEP, ATM, CN, TE, NA, CIP, SXT, and F	6	1 (5.8)

2	AMP, AMC, CTX, CAZ, CRO, FEP, ATM, CN, TE, NA, CIP, and SXT	5	1 (5.8)

3	AMP, AMC, FOX, CTX, CAZ, CRO, FEP, ATM, TE, NA, CIP, and SXT	4	2 (11.7)

4	AMP, AMC, FOX, CTX, CAZ, FEP, AK, TE, and SXT	4	1 (5.8)

5	AMP, AMC, FOX, CTX, CAZ, CRO, FEP, ATM, CN, NA, and CIP	3	1 (5.8)

6	AMP, AMC, FOX, CTX, CAZ, CRO, FEP, ATM, TE, and SXT	3	2 (11.7)

7	AMP, AMC, FOX, CAZ, TE, and SXT	3	1 (5.8)

## Data Availability

Data and materials are available to other researchers upon request.
